# Mutations in *OsDET1*, *OsCOP10*, and *OsDDB1* confer embryonic lethality and alter flavonoid accumulation in Rice (*Oryza sativa* L.) seed

**DOI:** 10.3389/fpls.2022.952856

**Published:** 2022-07-25

**Authors:** Backki Kim, Yoonjung Lee, Ji-Young Nam, Gileung Lee, Jeonghwan Seo, Dongryung Lee, Yoo-Hyun Cho, Soon-Wook Kwon, Hee-Jong Koh

**Affiliations:** ^1^Department of Agriculture, Forestry and Bioresources, Research Institute for Agriculture and Life Sciences, and Plant Genomics and Breeding Institute, Seoul National University, Seoul, South Korea; ^2^Department of Crop Science, Konkuk University, Seoul, South Korea; ^3^Advanced Radiation Technology Institute, Korea Atomic Energy Research Institute, Jeongeup, South Korea; ^4^National Institute of Crop Science, Rural Development Administration, Wanju, South Korea; ^5^Division of Biological and Environmental Sciences and Engineering, King Abdullah University of Science and Technology, Thuwal, Saudi Arabia; ^6^SeedPia, Suwon, South Korea; ^7^Department of Plant Bioscience, College of Natural Resources and Life Science, Pusan National University, Milyang, South Korea

**Keywords:** *yellowish-pericarp embryo lethal* (*yel*) mutant, *OsDET1*, CDD complex, embryo development, CRISPR/Cas9, rice (*Oryza sativa*)

## Abstract

Morphological and biochemical changes accompanying embryogenesis and seed development are crucial for plant survival and crop productivity. Here, we identified a novel *yellowish-pericarp embryo lethal* (*yel*) mutant of the *japonica* rice cultivar Sindongjin (*Oryza sativa* L.), namely, *yel*-*sdj*. Seeds of the *yel*-*sdj* mutant showed a yellowish pericarp and black embryo, and were embryonic lethal. Compared with wild-type seeds, the *yel*-*sdj* mutant seeds exhibited significantly reduced grain size, grain weight, and embryo weight, and a remarkably lower rate of embryo retention in kernels subjected to milling. However, the volume of air space between embryo and endosperm, density of embryo, and total phenolic content (TPC) and antioxidant activity of mature grains were significantly higher in the *yel*-*sdj* mutant than in the wild type. Genetic analysis and mapping revealed that the *yel*-*sdj* mutant was non-allelic to the *oscop1* null mutants *yel*-*hc*, *yel*-*cc*, and *yel*-*sk*, and its phenotype was controlled by a single recessive gene, *LOC*_*Os01g01484*, an ortholog of *Arabidopsis thaliana DE-ETIOLATED 1* (*DET1*). The *yel*-*sdj* mutant carried a 7 bp deletion in the second exon of *OsDET1*. Seeds of the *osdet1* knockout mutant, generated *via* CRISPR/Cas9-based gene editing, displayed the *yel* mutant phenotype. Consistent with the fact that OsDET1 interacts with CONSTITUTIVE PHOTOMORPHOGENIC 10 (OsCOP10) and UV-DAMAGED DNA BINDING PROTEIN 1 (OsDDB1) to form the COP10-DET1-DDB1 (CDD), seeds of *oscop10* and *osddb1* knockout mutants also showed the *yel* phenotype. These findings will enhance our understanding of the functional roles of *OsDET1* and the CDD complex in embryogenesis and flavonoid biosynthesis in rice seeds.

## Introduction

*DE-ETIOLATED 1* (*DET1*) encodes a nuclear-localized protein that presumably acts downstream of multiple photoreceptors to modulate the light-mediated signaling pathways (Pepper et al., 1994). DET1 plays an important role in regulating the expression of development-related genes and is highly conserved across higher eukaryotes (Schroeder et al., 2002). DET1 was first identified in dark-grown *Arabidopsis thaliana* seedlings, which showed a de-etiolated phenotype characterized by the expansion of leaves, inhibition of hypocotyl elongation, and accumulation of anthocyanins (Chory et al., 1989). Previously, genetic screening studies identified a class of mutants displaying de-etiolated or constitutive photomorphogenic phenotypes in the dark and uncovered that DET1 acts as a crucial regulator of light signaling during seedling development in *Arabidopsis* (Pepper et al., 1994; Mayer et al., 1996). Besides its function in seedling photomorphogenesis, DET1 plays an important role in the developmental and environmental responses of plants, as exemplified by its role in chloroplast development (Chory and Peto, 1990), circadian period regulation (Millar et al., 1995; Lau et al., 2011), flowering time regulation (Kang et al., 2015), UV tolerance (Castells et al., 2010), fruit pigmentation (Mustilli et al., 1999), and seed germination (Shi et al., 2015). Collectively, DET1 is a central regulator that integrates the light signal with various developmental and biosynthetic pathways in plants.

DET1 interacts with CONSTITUTIVE PHOTOMORPHOGENIC 10 (COP10) and UV-DAMAGED DNA BINDING PROTEIN 1 (DDB1) to form the COP10-DET1-DDB1 (CDD) complex, which acts as a ubiquitination-promoting factor to regulate photomorphogenesis in *Arabidopsis* (Schroeder et al., 2002; Yanagawa et al., 2004). Although the underlying molecular mechanism of the CDD complex has not been fully elucidated, some possible models for role of the CDD complex and its relationship have been established at the molecular level (see review for details, Lau and Deng, 2012). COP10, a ubiquitin-conjugating enzyme (E2) variant (UEV) protein, was identified as a negative regulator of photomorphogenic development in the dark (Wei et al., 1994; Suzuki et al., 2002). COP10 has the ability to enhance the activity of multiple E2 enzymes and directly interacts with both COP1 and the COP9 signalosome to mediate the repression of photomorphogenesis by degrading ELONGATED HYPOCOTYL 5 (HY5) (Osterlund et al., 2000; Suzuki et al., 2002; Yanagawa et al., 2004; Lau and Deng, 2009). DDB1, on the other hand, is highly conserved among eukaryotes and was originally identified in human as a recognition protein that counteracts UV-induced DNA damage, and plays a role in nucleotide excision repair (NER) (Chu and Chang, 1988). The *Arabidopsis* genome encodes two DDB1 homologs, DDB1a and DDB1b, which are 91% identical at the amino acid level (Schroeder et al., 2002). DDB1 functions as an adapter linking the substrate receptors to CULLIN4 (CUL4)-based E3 ligases for ubiquitination (He et al., 2006; Lee and Zhou, 2007). In *Arabidopsis*, COP10- or DDB1-containing complexes cooperate with CUL4 to form an E3 ligase machinery, which is involved in photomorphogenesis and ubiquitin-mediated protein degradation (Yanagawa et al., 2004; Chen et al., 2006; Ganpudi and Schroeder, 2013). However, unlike *Arabidopsis*, the molecular functions of COP10 and DDB1, not only as independent proteins but also as CDD complex components, remain unclear in rice.

In plants, successful embryogenesis is a prerequisite for proper seed germination and early vegetative growth. Furthermore, interaction between embryo and endosperm affects the agronomically important traits of plants, such as starch composition and endosperm size, which determine grain yield and quality (Lafon-Placette and Kohler, 2014; An et al., 2020). Thus, given the biological and agricultural importance of embryogenesis, the molecular mechanisms underlying this process have been of considerable interest. Over the last several decades, numerous embryonic lethal or embryo-defective mutants have been identified in the model dicot plant, *Arabidopsis* (Meinke and Sussex, 1979a,b), and in model monocots, maize (Clark and Sheridan, 1991; Sheridan and Clark, 1993) and rice (Nagato et al., 1989; Kitano et al., 1993; Hong et al., 1995). These mutants exhibit a wide range of phenotypes, such as no embryo, incomplete embryo organs, colorless embryo, albino, and pigmented cotyledons (Meinke and Sussex, 1979a; Meinke, 1985; Hong et al., 1995; Satoh et al., 1999). Among them, *Arabidopsis* “*fusca*” (*fus*) mutants display purple coloration in cotyledons, because of high-level anthocyanin accumulation, and exhibit seedling lethality and defective photomorphogenesis. Interestingly, some of the *fus* mutants we re revealed to be allelic to *cop/det* mutants exhibiting the “*fusca*” phenotype (Castle and Meinke, 1994; Misera et al., 1994). Previously, we reported the “*fusca*”-like *cop1* null mutants in rice, which we named as *yellowish*-*pericarp embryo lethal* (*yel*), and showed that the corresponding gene, *OsCOP1*, regulates flavonoid biosynthesis and embryo development. However, the effect of *COP1* mutation on embryo development and pigmentation in rice was different from that in *Arabidopsis*; unlike the *Arabidopsis fus* mutants, which exhibited seedling lethality and anthocyanin accumulation in the cotyledons, the rice *yel* mutants showed embryonic lethality and flavonoid accumulation in the embryo and pericarp (Kim et al., 2018, 2021).

In the present study, we characterized a novel rice *yel* mutant displaying yellowish-pericarp and embryonic lethality, and identified *OsDET1*, an ortholog of *Arabidopsis DET1*, as the causal gene. Furthermore, using the CRISPR/Cas9 gene editing tool, we confirmed that *oscop10* and *osddb1* knockout mutants also showed the *yel* phenotype. Although the role of *DET1* in photomorphogenic development has been extensively studied in *Arabidopsis*, limited information is available in rice. Therefore, characterization of the *osdet1*, *oscop10*, and *osddb1* null mutants conducted in this study provides new insights into the regulation of flavonoid biosynthesis and embryo development in rice.

## Materials and methods

### Plant materials and growth conditions

The novel *yel* mutant was derived from the *japonica* rice (*Oryza sativa* L. ssp. *japonica*) cultivar Sindongjin (SDJ) by gamma ray (γ-ray) irradiation, and named *yel-sdj*. Given its embryonic lethality, the *yel-sdj* mutant maintained as a heterozygote. An F_2_ mapping population was developed from a cross between the heterozygous *yel-sdj* mutant and a Korean *indica* rice accession, Milyang 23 (M.23). Additional F_2_ populations were derived by crossing the heterozygous *yel-sdj* mutant with SDJ (wild-type [WT]) and other *yel* mutants (*yel*-*hc*, *yel*-*cc*, and *yel*-*sk*) to calculate segregation ratios and perform the allelism test. The F_2_ populations as well as WT and *yel*-*sdj* mutant plants were cultivated in a paddy field at the Experimental Farm of Seoul National University, Suwon, South Korea. Knockout transgenic plants generated using the CRISPR/Cas9 technology were grown in the Living Modified Organism (LMO) experimental field (RDA-GA-AB-2011-014) at the Experimental Farm of Seoul National University, Suwon, South Korea.

### Weight measurements of dehulled grains, embryos, and endosperms

Rice grains were air-dried after harvesting, and moisture content was reduced to approximately 13%. Grains were stored in an environmentally controlled room at 10°C for 2 months, and then dehusked and hand-selected to eliminate cracked or abnormally developed seeds. The length, width, and thickness of a total of 90 mature dehulled rice grains (30 seeds × 3 replicates) of each genotype were measured using digimatic calipers (Mitutoyo, Japan). Hundred-grain, -endosperm, and -embryo weights (100 seeds × 3 replicates; 10% water content) were measured using an analytical balance (CAS Corporation, NJ, United States). To measure embryo and endosperm weight, embryos were excised from the grains and weighed separately. Phenotypic data collected from *yel*-*sdj* mutant and WT genotypes were statistically analyzed using SAS version 9.4 (SAS Institute Inc., Cary, NC, United States).

### Measurement of the rate of embryo retention in kernels

The rate of embryo retention in kernels was measured using a small-scale grain polisher (Kett, Tokyo, Japan). A total of 100 brown rice kernels, with uniform appearance, were selected from *yel*-*sdj* mutant and WT seeds, respectively, mixed, and polished together under the same milling conditions for 5 s. Subsequently, kernels completely devoid of the embryo and those with a retained embryo were counted for each genotype. Five replications were conducted and the average trait value was used for data analysis.

### Microcomputed tomography (CT) scan and image processing

Five dehulled kernels each of WT and *yel-sdj* mutant genotypes were randomly selected, and then scanned using SkyScan 1272 (Bruker, Belgium, Kontich), with pixel size set to 5 μm. The image acquisition process was carried out using the X-ray tube, with the following settings: voltage, 60 kV; current, 166 μA; exposure time, 0.45 s; four-frame averaging; rotation step, 0.40°; rotation angle, 180°. The scan duration was approximately 25 min. Following scanning, the raw images were converted to three-dimensional (3D) structures using the NRecon reconstruction software (SkyScan, Belgium), with the following settings: smoothing, 2; ring artifact correction, 24; beam hardening reduction, 50%. The resulting images were saved in bitmap (.bmp) format. The reconstructed images of the grains were then analyzed using 3D Slicer (v.4.13.0) (Fedorov et al., 2012).

### Extraction and sample preparation for biochemical analysis of wild-type and mutant rice seeds

To analyze the seed phenolic content and antioxidant activity, the extracts of WT and *yel*-*sdj* mutant seeds were prepared as described by Chung et al. (2017). Briefly, the seeds of each genotype were ground to a fine powder. Then, 1 g of each powdered sample was extracted with 10 mL of acetonitrile (ACN) and 2 mL of 0.1 N HCl, and sonicated using JAC-5020 Ultrasonic cleaner ABS (U1tech, Gyeonggi-Do, South Korea) at 40 kHz and room temperature for 20 min. After centrifugation at 1,962 × *g* and 4^°^C for 5 min, the supernatant was collected in a round-bottomed flask. The above process was repeated 3 and 13 times for WT and *yel*-*sdj* seed extracts, respectively. The final extracts were concentrated in a rotary vacuum evaporator (EYELA SB-1200; Tokyo Rikakikai Co., Ltd., Tokyo, Japan) at 35^°^C. The residue was reconstituted with 5 mL of 80% methanol and filtered through a 0.22 μm polytetrafluoroethylene (PTFE) syringe filter (CHOICE 13 mm; Thermo Scientific, Waltham, MA, United States).

### Total phenolic content measurement

Total phenolic content (TPC) was determined using a spectrophotometric assay based on the Lowry method, with slight modifications (Winters and Minchin, 2005). Briefly, 10 μL of each sample was mixed either with 990 μL of distilled water (blank) or with 990 μL of buffer (790 μL of distilled water, 50 μL of 1 N Folin-Ciocalteu reagent, and 150 μL of Na_2_CO_3_ in saturated NaOH solution). Following 1 h incubation at room temperature, absorbance was measured at 765 nm using a UV-Vis spectrophotometer. TPC was calculated from the calibration curve of gallic acid and expressed as micrograms of gallic acid equivalents per gram of dry weight (μg GAE/g DW). All samples were analyzed in triplicate.

### 2,2*-*diphenyl-1-picrylhydrazyl assay

Antioxidant enzyme activity in seeds was determined by performing the 2,2-diphenyl-1-picrylhydrazyl (DPPH) free-radical scavenging assay, as described previously (Kim et al., 2020). Briefly, 50 μL of the seed extract was added to 950 μL of 0.1 mM DPPH in methanol. The mixture was transferred to a 4 mL cuvette (10 mm × 10 mm × 45 mm; Ratiolab GmbH, Dreieich, Germany) and allowed to stand at room temperature in the dark for 30 min. Then, absorbance was measured at 517 nm using the OPTIZEN POP UV spectrophotometer (Mecasys Co., Daejeon, South Korea). The DPPH free-radical scavenging activity was calculated as inhibition percentage using the following equation:


$$Inhibition\left(\%\right)=\left[1-\left(OD_{s\,a\,m\,p\,l\,e}-OD_{c\,o\,n\,t\,r\,o\,l}\right)\right]\times 100$$

where OD_*sample*_ and OD_*control*_ represent the absorbance of the seed sample and DPPH standard solution, respectively.

### Map-based cloning

Genomic DNA was extracted from the *yel* mutant-type seeds and leaves of 549 F_2_ individuals derived from the *yel*-*sdj* × M.23 cross. To identify the gene responsible for the *yel*-*sdj* mutant phenotype, bulked segregant analysis (BSA) was performed using a set of single nucleotide polymorphism (SNP) markers developed previously by designing primers based on nucleotide sequence differences between *indica* and *japonica* rice accessions (Seo et al., 2020). To fine-map the *yel*-*sdj* locus, sequence-tagged site (STS) primers were designed with Primer3 (version 0.4.0),^1^ based on the available rice genome sequence data.^2^ Primers designed and used in this study are listed in Supplementary Table 1.

### Sequence analysis of *OsDET1*

Full-length sequence of the *OsDET1* gene was amplified from WT and *yel*-*sdj* mutant seeds by performing overlapping extension PCR. The amplified products were purified using a PCR purification kit (iNtRON Biotechnology, South Korea), cloned into the pGEM-T Easy Vector (Promega, United States), and transformed into *Escherichia coli* strain DH5α. The inserts were sequenced, and sequences were compared using the CodonCode Aligner software (version 1.6.3; CodonCode Corporation, MA, United States).

### CRISPR/Cas9 vector construction and rice transformation

To knock out the *OsDET1*, *OsCOP10*, and *OsDDB1* genes, CRISPR/Cas9 vectors were constructed as described previously (Lowder et al., 2015). Briefly, guide RNAs (gRNAs) targeting each gene were designed using web-based tools, CRISPR RGEN Tools^3^ (Park et al., 2015) and CRISPRdirect^4^ (Naito et al., 2015). *OsDET1* was targeted using two gRNAs, whereas *OsCOP10* and *OsDDB1* were each targeted using a single gRNA. The *OsDET1*-targeting gRNAs were cloned separately into two different gRNA expression vectors, pYPQ131C (Addgene plasmid #69284) and pYPQ132C (Addgene plasmid #69285), while *OsCOP10*- and *OsDDB1*-targeting gRNAs were cloned separately into pYPQ141C (Addgene plasmid #69292) under the expression of the *OsU6* promoter. The gRNA expression cassettes were then assembled into the Golden Gate recipient vector pYPQ142 (Addgene plasmid #69294). pYPQ165 (Addgene #109327) was used as a Cas9 entry vector, which contained an egg cell-specific promoter. Finally, a Gateway assembly LR reaction was performed using the Cas9 entry vector (pYPQ165), gRNA cassettes (pYPQ141C or pYPQ142), and pMDC99 binary vector to generate the T-DNA binary vectors.

The final constructs were transformed into the seeds of the *japonica* cultivar Dongjin *via Agrobacterium*-mediated transformation using the LBA4404 strain, as described previously (Nishimura et al., 2006), with slight modifications. Primers used for vector construction and genotyping are listed in Supplementary Table 1.

### RNA isolation and quantitative real-time PCR

All fresh plant samples were flash-frozen in liquid nitrogen. Total RNA was extracted from the leaf, leaf sheath, root, and young panicle (3 cm) of WT plants, and from 7-day-old seeds of both WT and *yel*-*sdj* mutant seeds, in three biological replicates, using TaKaRa MiniBEST Plant RNA Extraction Kit (Takara, Japan) according to the manufacturer’s instructions. Total RNA samples were subjected to first-strand cDNA synthesis using M-MLV reverse transcriptase (Promega, Madison, WI, United States), and qRT-PCR was performed using TB Green^®^ Premix Ex Taq™ II (Tli RNaseH Plus) (Takara Bio, Japan) on a CFX96™ Real-time PCR Detection System (Bio-Rad, Hercules, CA, United States) according to the manufacturer’s instructions. Primers used for qRT-PCR analysis are listed in Supplementary Table 1. Expression levels of genes were normalized relative to that of *ACTIN*, a housekeeping gene. Data were analyzed using the comparative Ct method. Expression levels were compared using two-tailed Student’s *t-*test.

## Results

### Morphological characterization of *yel-sdj* mutant seeds

**FIGURE 1:**
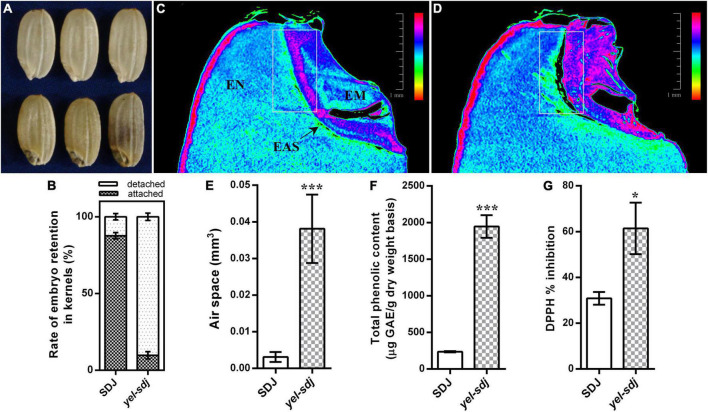
Comparison of the grain characteristics of wild-type (WT; Sindongjin [SDJ]) and *yel*-*sdj* mutant rice. **(A)** Evaluation of the morphology of WT (upper panel) and *yel*-*sdj* mutant (lower panel) grains. **(B)** Rate of embryo retention in kernels after milling for 5 s. Data represent the mean ± standard deviation (SD) of five biological replicates. **(C,D)** Longitudinal cross section of WT **(C)** and *yel*-*sdj* mutant **(D)** grains using computed tomography (CT). White rectangles indicate the area coinciding with the air space found between the embryo and endosperm (scale bar = 1 mm). The color scale represents embryo density. EN, endosperm; EM, embryo; EAS, endosperm adjacent to the scutellum. **(E–G)** Volume of air space between the embryo and endosperm **(E)**, content of total phenolics **(F)**, and antioxidant activity **(G)** in WT and *yel*-*sdj* mutant grains. Data represent the mean ± SD of three biological replicates. Asterisks indicate statistical significance, as determined by Student’s *t*-test (*p < 0.05, ***p < 0.001).

The novel *yel* mutant, *yel-sdj*, was derived by γ-ray irradiation of the *japonica* rice cultivar SDJ. The main distinctive feature of the *yel-sdj* mutant seed was its embryo and pericarp color; the embryo was black, and the pericarp was yellowish or mixed yellow-purple (Figure 1A). Homozygous *yel-sdj* mutant seeds failed to germinate in the standard germination test (data not shown). Additionally, the *yel*-*sdj* mutant grains exhibited significantly reduced length, width, and thickness compared with WT grains, although no significant difference was detected between the length-to-width ratio of dehulled *yel*-*sdj* mutant and WT grains (Table 1). Hundred-grain weight was significantly lower in *yel*-*sdj* than in the WT. Consistently, the hundred-endosperm and -embryo weights were also significantly lower in the *yel-sdj* mutant than in the WT (Table 1). These results indicate that the overall development of seed is affected in the *yel*-*sdj* mutant, resulting in altered pigmentation of embryo and pericarp, and reduced weight of grain, endosperm, and embryo.

**TABLE 1:** Measurement of grain related traits in the wild-type (WT) rice cultivar Sindongjin (SDJ) and *yel-sdj* mutant.

	Trait measurements						
Genotype	Grain length (mm)	Grain width (mm)	Grain length/width ratio	Grain thickness (mm)	100 kernel weight (g)	100 endosperm weight (g)	100 embryo weight (g)
SDJ	6.09 ± 0.168	3.20 ± 0.039	1.91 ± 0.045	2.01 ± 0.065	2.68 ± 0.025	2.60 ± 0.024	0.083 ± 0.001
*yel*-*sdj*	5.94 ± 0.158*	3.07 ± 0.074**	1.94 ± 0.087	1.92 ± 0.050**	2.46 ± 0.036**	2.40 ± 0.036**	0.064 ± 0.001**

Data represent mean ± standard deviation (SD). Asterisks indicate significant differences relative to the WT (*p < 0.05, **p < 0.01).

### Physical and histological properties of *yel-sdj* grains

While detaching embryos from grains to measure the embryo weight, we empirically found that the removal of *yel*-*sdj* embryos from grains was easier than that of WT embryos. Therefore, we investigated the rate of embryo retention in the kernels using a small-scale grain polisher. The results revealed that the rate of embryo retention was remarkably lower in the *yel*-*sdj* mutant than in the WT; while approximately 88% of WT kernels contained embryos after milling for 5 s, only 10% of *yel*-*sdj* kernels retained the embryos (Figure 1B). To understand why the strength of embryo attachment differed between *yel*-*sdj* and WT seeds, we examined the histological properties and internal morphology of WT and *yel*-*sdj* grains by CT. Interestingly, significantly greater volume of air space was observed between the embryo and endosperm in *yel*-*sdj* mutant seeds than in WT seeds (Figures 1C,D and Supplementary File 1). The volume of air space between the embryo and endosperm in the *yel*-*sdj* mutant (0.0381 ± 0.00834 mm^3^) was approximately 12 times higher than that in the WT (0.0031 ± 0.00121 mm^3^) (Figure 1E). In addition, the density of embryo was relatively higher, and the area of endosperm adjacent to the scutellum (EAS) was wider in the *yel*-*sdj* mutant than in the WT (Figures 1C,D). These results suggest that the formation of air space along the border between the embryo and endosperm is responsible for the easy detachment of embryo from the kernel in the *yel*-*sdj* mutant.

### Chemical properties of *yel-sdj* mutant seeds

Seed TPC and antioxidant activity were assessed in the *yel*-*sdj* mutant and WT using grain extracts. The TPC of *yel*-*sdj* grains (1,946 μg GAE/g DW) was approximately eightfold higher than that of WT grains (236 μg GAE/g DW), a significant difference (*p* < 0.001) (Figure 1F). Additionally, the DPPH radical scavenging activity in *yel*-*sdj* grains (61.4% inhibition) was approximately twofold higher than that in WT grains (30.8% inhibition) (*p* < 0.05; Figure 1G). These results suggest that the high TPC of *yel*-*sdj* grains leads to increased antioxidant activity.

### Genetic analysis of the *yel-sdj* mutant

Since the homozygous *yel*-*sdj* mutant was embryo lethal, we crossed a heterozygous *yel*-*sdj* mutant plant with the WT cultivar SDJ and used the resultant F_1_ and F_2_ populations for genetic analysis. The F_2_ seeds showed a WT:*yel*-*sdj* segregation ratio of 3:1 (Table 2). Additionally, to determine whether the *yel*-*sdj* mutant allele is novel, we conducted an allelism test by crossing the *yel*-*sdj* mutant with three *oscop1* null mutants, *yel*-*hc*, *yel*-*cc*, and *yel*-*sk*. Since homozygous *oscop1* mutants are embryo lethal (like the *yel*-*sdj* mutant), heterozygous plants of each *oscop1* mutant were used in these crosses. All F_1_ seeds obtained from the three crosses showed WT phenotype, and the F_2_ seeds of only some F_1_ plants showed a WT:*yel* segregation ratio of 9:7 (Table 2). Therefore, we conclude that the *yel*-*sdj* mutant phenotype was controlled by a single recessive gene, and *yel*-*sdj* and *oscop1* null mutants (*yel*-*hc*, *yel*-*sk*, and *yel*-*cc*) were non-allelic, indicating that a novel locus is responsible for the *yel*-*sdj* phenotype.

**TABLE 2:** Genetic analysis of the *yel*-*sdj* mutant.

	No. of F_2_ grains						
Cross	Normal phenotype	*yel* phenotype	Total	df	Expected ratio	χ^2^	*P*-value
*yel*-*sdj* x Sindongjin	559	176	735	1	3:1	0.44	0.509
*yel*-*hc* x *yel*-*sdj* -5	254	185	439	1	9:7	0.40	0.528
*yel*-*sk* x *yel*-*sdj* -3	321	264	585	1	9:7	0.40	0.529
*yel*-*cc* x *yel*-*sdj* -2	289	203	492	1	9:7	1.14	0.286

### Map-based cloning of the gene responsible for the *yel-sdj* phenotype

**FIGURE 2:**
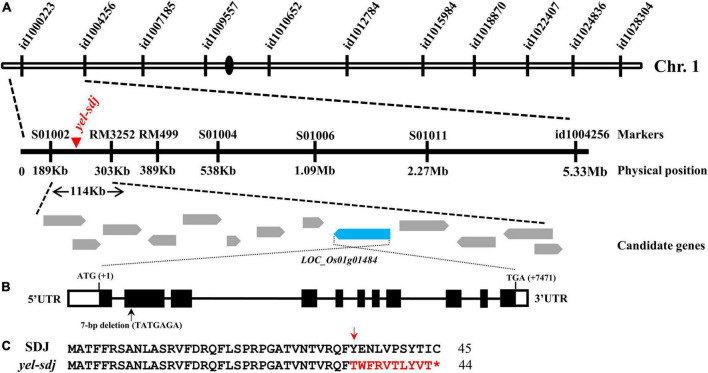
Map-based cloning of the gene responsible for the *yel-sdj* mutant phenotype. **(A)** Schematic showing the physical position of the causal locus on rice chromosome 1, as identified by map-based cloning. **(B)** Gene structure of *OsDET1*. Black lines, white solid boxes, and black solid boxes indicate introns, untranslated regions, and exons, respectively. The 7 bp deletion is indicated with a black arrow. ATG and TGA indicate the initiation and termination codons, respectively. **(C)** Comparison of the predicted amino acid sequence of the mutated region of gene between the WT (SDJ) and *yel*-*sdj* mutant. The 7 bp deletion resulted in a frameshift (red arrow) and premature stop (red asterisk) in *yel*-*sdj*. Amino acids in the frameshift region are indicated in red.

An F_2_ population derived from a cross between a *yel*-*sdj* heterozygous mutant plant and M.23 was used to map the locus responsible for the *yel*-*sdj* phenotype. To conduct preliminary genetic mapping, BSA was performed using the 96 SNP array, and the *yel*-*sdj* locus was mapped to a region between the start of chromosome 1 and id1004256 (Figure 2A). To refine the flanking region, an F_2_ population of 549 individuals was genotyped using newly designed markers (Supplementary Table 1). Finally, the *yel*-*sdj* locus was mapped to an approximately 114 kb region between the S01002 and RM3252 markers, and 13 candidate genes were identified within this region (Figure 2A). Among these 13 genes, *LOC*_*Os01g01484* (*Os01g0104600*), an ortholog of *AtDET1*, was selected as a strong candidate gene associated with the *yel-sdj* phenotype, given its previously reported role in embryonic lethality and anthocyanin accumulation in *Arabidopsis*. Sequence analysis in SDJ and *yel*-*sdj* mutant revealed a deletion of 7 bp (TATGAGA, where the A of ATG is +1 bp) at position +365 to +371 bp in the second exon of locus *LOC*_*Os01g01484* in the *yel*-*sdj* mutant (Figure 2B). The 7 bp deletion was predicted to cause a frameshift and consequently a premature stop codon at the 45th amino acid, resulting in aberrant protein production (Figure 2C).

### CRISPR/Cas9-based validation of the mutation causing the *yel-sdj* phenotype

**FIGURE 3:**
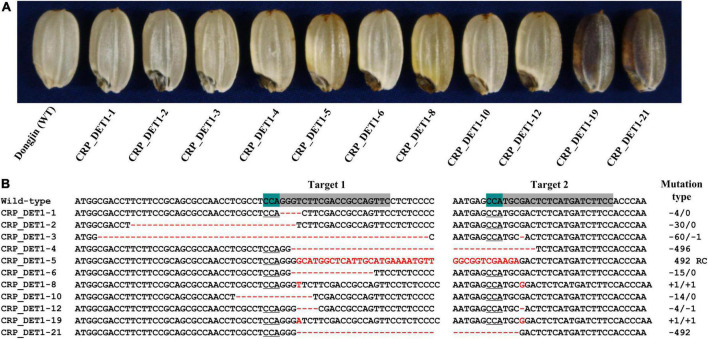
Grain phenotypic analysis and sequence analysis of CRISPR/Cas9-induced knockout *yel* mutants generated by targeting the *OsDET1* gene. **(A)** Grain phenotype of Dongjin (WT) and transgenic seeds. Grains showing the *yel* phenotype were randomly selected from each positive T_0_ transgenic plant. **(B)** Comparison of the *OsDET1* nucleotide sequence targeted by CRISPR/Cas9 in WT and mutant plants. Targets 1 and 2 represent the first and second exons, respectively, of *OsDET1* (*LOC*_*Os01g01484*/*Os01g0104600*). Red dashes and letters indicate deletions and insertions, respectively, in transgenic lines. The protospacer adjacent motif (PAM) is highlighted in green in the WT and is underlined in mutant lines. Sequences of targets 1 and 2 are highlighted in gray in the WT. Mutation types are shown to the right of each mutated sequence (-, deletion; +, insertion; RC, reverse complementary).

To confirm the association of *OsDET1* with the *yel* phenotype, the protein-coding sequence of this gene was edited using the CRISPR/Cas9 system. Two gRNAs complimentary to the sequence located near the 7 bp deletion were designed to target the coding sequence of *OsDET1*, and an egg cell-specific Cas9 promoter was used for vector construction to overcome lethality during tissue culture in the homozygous T_0_ plant regeneration. A total of 24 positive T_0_ transgenic plants were obtained, and T_1_ seeds exhibiting the *yel* mutant phenotype were identified, although these seeds showed variable pericarp color (Figure 3A). Mutations in target regions in T_1_ seeds displaying the *yel* mutant phenotype were confirmed by PCR and Sanger sequencing. Sequence analysis revealed a variety of insertions and deletions at the two target sites in all *yel* phenotype seeds. Target site 1 (gRNA1) showed relatively higher frequency of mutations than target site 2 (gRNA2) (Figure 3B). All insertions and deletions introduced into the *OsDET1* gene in *yel* phenotype seeds were predicted to lead to frameshift mutations and premature stop codons. These results demonstrated that the *yel* mutant phenotype was caused by the loss-of-function of *OsDET1*. Additionally, embryo development was severely compromised in the *osdet1* null mutant, indicating that *OsDET1* is essential for maintaining normal embryogenesis in rice.

### Targeted mutagenesis of *OsCOP10* and *OsDDB1*

**FIGURE 4:**
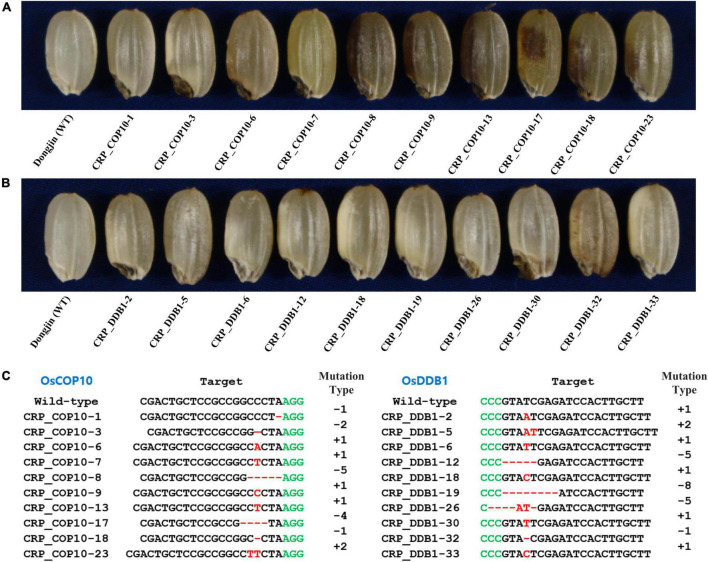
Grain phenotypic analysis and sequence analysis of CRISPR/Cas9-induced knockout *yel* mutants generated by targeting *OsCOP10* and *OsDDB1* genes. **(A,B)** Grain phenotype of Dongjin (WT) seeds and CRISPR mutant seeds generated by targeting *OsCOP10* **(A)** and *OsDDB1* **(B)**. Grains showing the *yel* phenotype were randomly selected from each positive T_0_ transgenic plant. **(C)** Sequence comparison of *OsCOP10* (*LOC*_*Os07g38940*/*Os07g0577400*) and *OsDDB1 (LOC*_*Os05g51480*/*Os05g0592400*) target regions in the WT and mutants. The first exon of *OsCOP10* and second exon of *OsDDB1* were targeted to induce mutations. Red dashes and letters indicate deletions and insertions, respectively, in transgenic lines. Black and green letters indicate the target sequence and PAM, respectively. Mutation types are shown to the right of each mutated sequence (-, deletion; +, insertion).

To determine if the genes encoding OsCOP10 and OsDDB1, which form the CDD complex together with OsDET1, are also involved in embryo development and flavonoid biosynthesis in rice, we mutated *OsCOP10* and *OsDDB1* using the CRISPR/Cas9 technology. CRISPR/Cas9 vectors designed to target the first exon of *OsCOP10* (*LOC_Os07g38940*/*Os07g0577400*) or second exon of *OsDDB1* (*LOC_Os05g51480*/*Os05g0592400*) were used for rice transformation, and seeds appearing phenotypically similar to those with the *yel* phenotype were collected from T_0_ positive transgenic plants (Figures 4A,B). Analysis of sequences targeted by *OsCOP10*- and *OsDDB1*-specific gRNAs revealed that all seeds exhibiting the *yel* phenotype carried mutations at the target sites (Figure 4C). This result indicates that loss-of-function mutations of *OsCOP10* and *OsDDB1* result in embryo lethality and altered pigmentation of the embryo and pericarp. Furthermore, this result implies that OsDET1 associates with OsCOP10 and OsDDB1 to form the CDD complex, and genes encoding all three proteins participate together in pathways regulating embryogenesis and flavonoid biosynthesis in rice.

### Expression analysis of *OsDET1*

**FIGURE 5:**
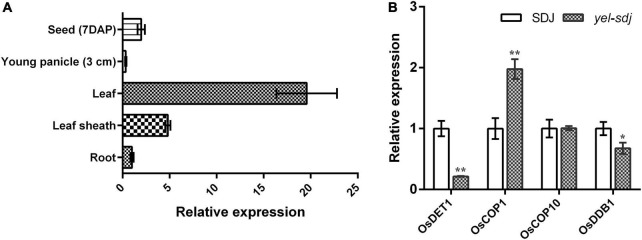
Expression analysis of *OsDET1* and *yel* phenotype-associated genes. **(A)** Quantitative real-time PCR (qRT-PCR) analysis of *OsDET1* in various organs of SDJ (WT). **(B)** Relative expression levels of *yel* phenotype-associated genes in the developing seeds of SDJ (WT) and *yel*-*sdj* mutant at 7 days after pollination (DAP). Expression level of genes was normalized relative to that of *ACTIN*. Data represent mean ± SD of three biological replicates. Asterisks indicate statistical significance, as determined by Student’s *t*-test (*p < 0.05, **p < 0.01).

The expression pattern of *OsDET1* in different plant organs was investigated by qRT-PCR. We found that *OsDET1* was predominantly expressed in the leaf and relatively higher in the leaf sheath and developing seed than in the root and young panicle in WT plants (Figure 5A). Since the *yel* phenotype was observed in seeds carrying mutations in photomorphogenesis-related genes, we examined the expression levels of genes encoding the CDD complex components and *OsCOP1* in *yel*-*sdj* mutant seeds at 7 days after pollination (DAP). The relative expression levels of *OsDET1* and *OsDDB1* were lower, whereas that of *OsCOP1* was significantly higher in developing *yel*-*sdj* mutant seeds than in WT seeds. No significant difference was detected in expression level of *OsCOP10* between the WT and *yel*-*sdj* mutant (Figure 5B).

## Discussion

DET1, a key negative regulator of light signaling, has been extensively studied as a repressor of photomorphogenesis, together with COP1, in *Arabidopsis*. However, unlike in *Arabidopsis*, only a few *det1* mutants have been reported in rice. Genetic complementation analysis demonstrated that a single nucleotide mutation in *OsDET1* can increase the leaf chlorophyll content in rice (Huang et al., 2013). Furthermore, *OsDET1* influences rice seed germination and seedling growth, and triggers dark-induced leaf senescence, by modulating the signaling pathway and biosynthesis of abscisic acid (ABA) (Zang et al., 2016). In the present study, we identified a novel *det1* null mutant of rice that displayed yellow and black pigmentation in the pericarp and embryo, respectively, and exhibited embryonic lethality (Figure 1A). The *yel*-*sdj* mutant, unlike previously reported rice mutants harboring a weak allele or transgenic rice plants generated by RNA interference (RNAi), is likely to be complete a loss-of-function mutant, based on the severity of its phenotype. This presumption is supported by the *yel* mutant phenotype of T_1_ transgenic seeds of the *osdet1* knockout mutant generated using the CRISPR/Cas9 system (Figure 3). Furthermore, severe developmental defects, leading to seedling lethality and failure to germinate, have been observed in *OsDET1* RNAi transgenic plants, which expression level was reduced markedly at the vegetative growth stage and T_1_ transgenic seeds, respectively (Zang et al., 2016). These results indicate that *yel*-*sdj* is a null mutant, and its *yel* phenotype is caused by the loss-of-function of *OsDET1.* In addition, we previously identified three *Arabidopsis* “*fusca*”-like *cop1* null mutants (*yel*-*hc*, *yel*-*sk*, and *yel*-*cc*) in rice. These mutants carry loss-of-function alleles of *OsCOP1* and share several phenotypic characteristics with the *yel*-*sdj* mutant, including embryonic lethality and high-level flavonoid accumulation in the pericarp and embryo (Kim et al., 2021). Similarly, the *Arabidopsis det1* null mutant displays the phenotypic characteristics of strong *cop1* mutant alleles, such as short hypocotyls, opened cotyledons, and anthocyanin accumulation (McNellis et al., 1994; Misera et al., 1994; Pepper et al., 1994). Thus, the phenotype of the *osdet1* null mutant is similar to that of *oscop1* null mutants, which implies a molecular link between OsDET1 and OsCOP1, both of which are involved in the regulation of embryo development and flavonoid biosynthesis in rice.

The role of the CDD complex in plants has been discovered through mutant screens. In *Arabidopsis*, both DET1 and COP10 have been identified as one of the pleiotropic COP/DET/FUS loci that act to repress photomorphogenic development of seedlings in the dark. Additionally, the *cop10*-*1* and *det1*-*6* T-DNA insertion mutants exhibit seedling lethality and *fus* phenotypic characteristics (such as anthocyanin accumulation) (Pepper et al., 1994; Wei et al., 1994). Consistent with these observations, complementation tests determined that *det1* and *cop10* are allelic to *fus2* and *fus9*, respectively (Castle and Meinke, 1994; Misera et al., 1994). However, mutations in *DDB1*, which encodes another component of the CDD complex, result in different phenotypic changes compared with mutations in *DET1* and *COP10*. For example, the *ddb1a* null mutant shows no obvious phenotype, whereas the *det1-1 ddb1a* double mutant exhibits enhanced *det1* null mutant phenotype. By contrast, the loss-of-function *ddb1b* mutants exhibit both embryo lethal and viable phenotypes (Schroeder et al., 2002; Bernhardt et al., 2010). In tomato (*Solanum lycopersicum*), the *High Pigment* (*HP*) genes, *HP1* (Liu et al., 2004) and *HP2* (Mustilli et al., 1999), encode DDB1 and DET1 homologs of *Arabidopsis*, respectively; however, the phenotypic characteristics of tomato *hp1* and *hp2* mutants differ from those of *Arabidopsis ddb1* and *det1* mutants, respectively. Tomato *hp1* and *hp2* mutants show no obvious phenotype in the dark; however, when grown under light, both display high-level anthocyanin accumulation in seedlings, short, and dark plants, dark-green immature fruits (due to the overproduction of chlorophyll), and increased flavonoid and carotenoid production in ripe fruits (Yen et al., 1997; Mustilli et al., 1999; Liu et al., 2004). The phenotypes of *LeCOP1LIKE* RNAi seedlings and fruits are similar to those of light-grown *hp1* and *hp2* mutants (Mustilli et al., 1999; Liu et al., 2004). Besides phenotypic similarities among the mutants of CDD components, the evidence that the genes forming CDD complex are associated with each other has been revealed at the molecular level. *OsDET1* interacts physically with *OsDDB1* and *OsCOP10* in rice. (Zang et al., 2016), and DET1 and DDB1 interact with each other to suppress photomorphogenesis in *Arabidopsis* (Schroeder et al., 2002). In addition, COP10 interacts with COP1 to promote the degradation of photomorphogenesis-regulating proteins in *Arabidopsis* (Suzuki et al., 2002; Yanagawa et al., 2004). In this study, we demonstrated that all seeds displaying the *yel* phenotype carried mutations in *OsDET1*, *OsCOP10*, and *OsDDB1* genes (Figures 1A, 3A, 4A,B). Given the phenotype of our knockout mutants, this result implies that genes encoding the CDD components in rice, *OsDET1*, *OsCOP10*, and *OsDDB1*, are essential for the functional roles or share molecular pathways or genetic signals during embryo development and flavonoid biosynthesis. Furthermore, the *osdet1* null mutant was phenotypically highly similar to the *oscop1* null mutants (*yel*-*hc*, *yel*-*cc*, and *yel*-*sk*), although COP1 and DET1 seem to regulate ubiquitination independently through distinct multimeric units. This result suggests the possibility that the CDD complex functions together with COP1 to regulate flavonoid biosynthesis and embryogenesis in rice. These findings suggest that *OsDET1*, *OsCOP10*, *OsDDB1*, and *OsCOP1* perform a common function, and therefore the loss-of-function mutation of any of these genes results in the *yel* phenotype in rice seeds. Taken together, these findings explain why different *yel* mutants, harboring mutations in different genes, exhibit phenotypic similarities.

In *Arabidopsis*, COP1 is a RING E3 ubiquitin ligase that works in complex with SUPPRESSOR OF PHYA-105 (SPA1) protein, and the COP1/SPA1 complex targets photomorphogenic-promoting transcription factors, such as HY5, HY5 HOMOLOG (HYH), LONG HYPOCOTYL IN FAR-RED 1 (HFR1) for ubiquitination, and protein degradation in the dark (Hoecker and Quail, 2001; Holm et al., 2002; Jang et al., 2005). Furthermore, the CDD complex, consisting of DET1, COP10, and DDB1, acts together with the COP1 E3 ligase complex for the COP1-mediated protein degradation (Osterlund et al., 2000; Yanagawa et al., 2004; Canibano et al., 2021). In addition, the CDD complex forms CUL4-CDD E3 ubiquitin ligase through DDB1 and enhances E3 activity, which is required for the degradation of key regulators and other substrates (Chen et al., 2006; Lau and Deng, 2012). Among the photomorphogenesis-promoting transcription factors, it is well known that HY5, a bZIP transcription factor, is a central regulator of photomorphogenesis and positively regulates anthocyanin biosynthesis by binding transcription factors, such as *PRODUCTION OF ANTHOCYANIN PIGMENT1* (*PAP1*), *MYB12*, *MYB111*, which further activate the regulatory genes and structural genes (Holm et al., 2002; Stracke et al., 2010; Gangappa and Botto, 2016). However, unlike *Arabidopsis*, little is known about the role of *OsDET1*, *OsCOP10*, and *OsDDB1*, which affect flavonoid biosynthesis in rice. Thus, the possible mechanism could be that the *OsDET1*, *OsCOP10*, and *OsDDB1* mutations inhibit HY5 ubiquitination and degradation, and the resulting *yel* phenotype may support the transcriptional regulatory role where the COP1 and CDD complex negatively regulates HY5, a positive regulator of flavonoid biosynthesis. Namely, upregulated HY5 activates transcription factors which regulates flavonoid biosynthesis genes, resulting in flavonoid accumulation in embryo and pericarp of rice grain. The molecular function and mechanism of *OsDET1*, *OsCOP10*, and *OsDDB1*, CDD complex components, in flavonoid biosynthesis still remain unclear in rice. Therefore, further efforts are required to better understand the role of the CDD complex in flavonoid biosynthesis including embryo development.

Interaction between the embryo and endosperm affects not only the growth of embryo and endosperm itself but also seed development. It has been reported that maize embryo is separated from the endosperm by fibrous layer and EAS (Doll and Ingram, 2022). In particular, the EAS, which originates from the starchy endosperm cell layer adjacent to the scutellum, is the region where the cell death and the accumulation of crushed cell walls happen, allowing the embryo expansion as the embryo grows toward the endosperm (Doll et al., 2020; Doll and Ingram, 2022). In addition, transcriptome analysis revealed that the genes involved in sugar and amino acid transport such as the SWEET family and UMAMIT family genes are strongly activated in the EAS to provide nutrition to the embryo (Doll et al., 2020). In the present study, we found that the embryo of *yel*-*sdj* mutant is easily detached from the kernels (Figure 1B), and micro-CT analysis revealed that a dramatically wide area of EAS was observed in matured *yel*-*sdj* mutant seed (Figures 1C,D). Although the molecular mechanism associated with the development of EAS by the mutation of *OsDET1* is still unclear, it may be assumed that the degradation of starch or cell death in the broad EAS region reduced EAS density, resulting in loosening of the starch granules packing and embryo-endosperm interface tissue. Subsequently, as the seeds mature, its water content decreases thus, embryo split from the adjacent endosperm and forms air space between embryo and endosperm. This abnormal embryo-endosperm adhesion enables the embryo of *yel*-*sdj* to detach easily from the kernels. Taken together, the elucidation of *OsDET1* function, which affects EAS formation, will provide novel insights into seed development, especially the embryo-endosperm interaction of monocots, and expand our understanding of the molecular mechanisms during embryogenesis in higher plants.

Overall, we identified a novel mutant (*yel*-*sdj*) exhibiting yellowish pericarp and embryonic lethality, and showed that *OsDET1* plays a crucial role in flavonoid biosynthesis and embryogenesis in rice seed. In addition, we demonstrated that mutations in *OsCOP10* and *OsDDB1*, which encode members of the CDD complex, cause phenotypes similar to those exhibited by the typical *yel* mutants, such as *yel*-*hc*, *yel*-*cc*, *yel*-*sk*, and *yel*-*sdj*. Additionally, our results demonstrated that modification of the light signal transduction machinery could have a significant effect on flavonoid biosynthesis and embryo development in rice seed. Further examination of mutations in other light signal transduction machinery genes, whose proteins associate with OsDET1 and OsCOP1, will facilitate a better understanding of the common molecular mechanisms and metabolic pathways involved in embryo development and flavonoid biosynthesis in rice seed.

## Data availability statement

The original contributions presented in this study are included in the article/Supplementary Material, further inquiries can be directed to the corresponding author/s.

## Author contributions

BK designed and performed the research, analyzed the data, and wrote the manuscript. Y-HC provided the material. BK, YL, J-YN, GL, and JS carried out experiments. DL validated the data. S-WK designed and supervised the experiment. H-JK designed and supervised the experiment and revised the manuscript. All authors have read and agreed to the final version of the manuscript.

## Footnotes

1
http://frodo.wi.mit.edu/primer3

2
http://www.ncbi.nlm.nih.gov

3
http://www.rgenome.net/cas-designer/

4
https://crispr.dbcls.jp/

## References

[B1] An L. Tao Y. Chen H. He M. Xiao F. Li G. (2020). Embryo-endosperm interaction and its agronomic relevance to rice quality. *Front. Plant Sci.* 11:587641. 10.3389/fpls.2020.587641 33424883 PMC7793959

[B2] Bernhardt A. Mooney S. Hellmann H. (2010). Arabidopsis DDB1a and DDB1b are critical for embryo development. *Planta* 232 555–566. 10.1007/s00425-010-1195-9 20499085

[B3] Canibano E. Bourbousse C. Garcia-Leon M. Garnelo Gomez B. Wolff L. Garcia-Baudino C. (2021). DET1-mediated COP1 regulation avoids HY5 activity over second-site gene targets to tune plant photomorphogenesis. *Mol. Plant* 14 963–982. 10.1016/j.molp.2021.03.009 33711490

[B4] Castells E. Molinier J. Drevensek S. Genschik P. Barneche F. Bowler C. (2010). det1-1-induced UV-C hyposensitivity through UVR3 and PHR1 photolyase gene over-expression. *Plant J.* 63 392–404. 10.1111/j.1365-313X.2010.04249.x 20487384

[B5] Castle L. A. Meinke D. W. (1994). A FUSCA gene of Arabidopsis encodes a novel protein essential for plant development. *Plant Cell* 6 25–41. 10.1105/tpc.6.1.25 8130643 PMC160413

[B6] Chen H. Shen Y. Tang X. Yu L. Wang J. Guo L. (2006). *Arabidopsis* CULLIN4 Forms an E3 ubiquitin ligase with RBX1 and the CDD complex in mediating light control of development. *Plant Cell* 18 1991–2004. 10.1105/tpc.106.043224 16844902 PMC1533989

[B7] Chory J. Peto C. Feinbaum R. Pratt L. Ausubel F. (1989). Arabidopsis thaliana mutant that develops as a light-grown plant in the absence of light. *Cell* 58 991–999. 10.1016/0092-8674(89)90950-1 2776216

[B8] Chory J. Peto C. A. (1990). Mutations in the DET1 gene affect cell-type-specific expression of light-regulated genes and chloroplast development in *Arabidopsis*. *Proc. Natl. Acad. Sci. U.S.A.* 87 8776–8780. 10.1073/pnas.87.22.8776 2247447 PMC55042

[B9] Chu G. Chang E. (1988). Xeroderma pigmentosum group E cells lack a nuclear factor that binds to damaged DNA. *Science* 242 564–567. 10.1126/science.3175673 3175673

[B10] Chung I. M. Oh J. Y. Kim S. H. (2017). Comparative study of phenolic compounds, vitamin E, and fatty acids compositional profiles in black seed-coated soybeans (*Glycine Max* (L.) Merrill) depending on pickling period in brewed vinegar. *Chem. Cent. J.* 11 64. 10.1186/s13065-017-0298-9 29086850 PMC5515724

[B11] Clark J. K. Sheridan W. F. (1991). Isolation and characterization of 51 embryo-specific Mutations of Maize. *Plant Cell* 3 935–951. 10.1105/tpc.3.9.935 12324623 PMC160061

[B12] Doll N. M. Ingram G. C. (2022). Embryo-Endosperm Interactions. *Annu. Rev. Plant Biol.* 73 293–321. 10.1146/annurev-arplant-102820-091838 35130443

[B13] Doll N. M. Just J. Brunaud V. Caius J. Grimault A. Depege-Fargeix N. (2020). Transcriptomics at Maize Embryo/Endosperm Interfaces Identifies a Transcriptionally Distinct Endosperm Subdomain Adjacent to the Embryo Scutellum([OPEN]). *Plant Cell* 32 833–852. 10.1105/tpc.19.00756 32086366 PMC7145466

[B14] Fedorov A. Beichel R. Kalpathy-Cramer J. Finet J. Fillion-Robin J. C. Pujol S. (2012). 3D Slicer as an image computing platform for the Quantitative Imaging Network. *Magn. Reson. Imaging* 30 1323–1341. 10.1016/j.mri.2012.05.001 22770690 PMC3466397

[B15] Gangappa S. N. Botto J. F. (2016). The multifaceted roles of HY5 in plant growth and development. *Mol. Plant* 9 1353–1365. 10.1016/j.molp.2016.07.002 27435853

[B16] Ganpudi A. L. Schroeder D. F. (2013). Genetic interactions of Arabidopsis thaliana damaged DNA binding protein 1B (DDB1B) with DDB1A, DET1, and COP1. *G3* 3 493–503. 10.1534/g3.112.005249 23450167 PMC3583456

[B17] He Y. Z. J. Mccall C. M. Hu J. Zeng Y. X. Xiong Y. (2006). DDB1 functions as a linker to recruit receptor WD40 proteins to CUL4-ROC1 ubiquitin ligases. *Genes Dev.* 20 2949–2954. 10.1101/gad.1483206 17079684 PMC1620025

[B18] Hoecker U. Quail P. H. (2001). The phytochrome A-specific signaling intermediate SPA1 interacts directly with COP1, a constitutive repressor of light signaling in *Arabidopsis*. *J. Biol. Chem.* 276 38173–38178. 10.1074/jbc.M103140200 11461903

[B19] Holm M. Ma L. G. Qu L. J. Deng X. W. (2002). Two interacting bZIP proteins are direct targets of COP1-mediated control of light-dependent gene expression in *Arabidopsis*. *Genes Dev.* 16 1247–1259. 10.1101/gad.969702 12023303 PMC186273

[B20] Hong S. K. Aoki T. Kitano H. Satoh H. Nagato Y. (1995). Phenotypic diversity of 188 Rice Embryo Mutants. *Dev. Genet.* 16 298–310. 10.1002/dvg.1020160403

[B21] Huang J. Qin F. Zang G. Kang Z. Zou H. Hu F. (2013). Mutation of OsDET1 increases chlorophyll content in rice. *Plant Sci.* 210 241–249. 10.1016/j.plantsci.2013.06.003 23849131

[B22] Jang I. C. Yang J. Y. Seo H. S. Chua N. H. (2005). HFR1 is targeted by COP1 E3 ligase for post-translational proteolysis during phytochrome A signaling. *Genes Dev.* 19 593–602. 10.1101/gad.1247205 15741320 PMC551579

[B23] Kang M. Y. Yoo S. C. Kwon H. Y. Lee B. D. Cho J. N. Noh Y. S. (2015). Negative regulatory roles of DE-ETIOLATED1 in flowering time in *Arabidopsis*. *Sci. Rep.* 5:9728. 10.1038/srep09728 25962685 PMC4428065

[B24] Kim B. Piao R. Lee G. Koh E. Lee Y. Woo S. (2021). OsCOP1 regulates embryo development and flavonoid biosynthesis in rice (*Oryza sativa* L.). *Theor. Appl. Genet.* 134 2587–2601. 10.1007/s00122-021-03844-9 33950284 PMC8277627

[B25] Kim B. Woo S. Kim M. J. Kwon S. W. Lee J. Sung S. H. (2018). Identification and quantification of flavonoids in yellow grain mutant of rice (*Oryza sativa* L.). *Food Chem.* 241 154–162. 10.1016/j.foodchem.2017.08.089 28958514

[B26] Kim S. H. Yang Y. J. Chung I. M. (2020). The effect of degree of milling on the nutraceutical content in ecofriendly and conventional rice (*Oryza sativa* L.). *Foods* 9:1297. 10.3390/foods9091297 32942566 PMC7555660

[B27] Kitano H. Tamura Y. Satoh H. Nagato Y. (1993). Hierarchical regulation of organ differentiation during embryogenesis in rice. *Plant J.* 3 607–610. 10.1046/j.1365-313X.1993.03040607.x

[B28] Lafon-Placette C. Kohler C. (2014). Embryo and endosperm, partners in seed development. *Curr. Opin. Plant Biol.* 17 64–69. 10.1016/j.pbi.2013.11.008 24507496

[B29] Lau O. S. Deng X. W. (2009). Effect of *Arabidopsis* COP10 ubiquitin E2 enhancement activity across E2 families and functional conservation among its canonical homologues. *Biochem. J.* 418 683–690. 10.1042/BJ20081943 19061479

[B30] Lau O. S. Deng X. W. (2012). The photomorphogenic repressors COP1 and DET1: 20 years later. *Trends Plant Sci.* 17 584–593. 10.1016/j.tplants.2012.05.004 22705257

[B31] Lau O. S. Huang X. Charron J. B. Lee J. H. Li G. Deng X. W. (2011). Interaction of *Arabidopsis* DET1 with CCA1 and LHY in mediating transcriptional repression in the plant circadian clock. *Mol. Cell* 43 703–712. 10.1016/j.molcel.2011.07.013 21884973 PMC3204374

[B32] Lee J. Zhou P. (2007). DCAFs, the missing link of the CUL4-DDB1 ubiquitin ligase. *Mol. Cell* 26 775–780. 10.1016/j.molcel.2007.06.001 17588513

[B33] Liu Y. S. Roof S. Ye Z. B. Barry C. Van Tuinen A. Vrebalov J. (2004). Manipulation of light signal transduction as a means of modifying fruit nutritional quality in tomato. *Proc. Natl. Acad. Sci. U.S.A.* 101 9897–9902. 10.1073/pnas.0400935101 15178762 PMC470770

[B34] Lowder L. G. Zhang D. Baltes N. J. Paul J. W. III Tang X. Zheng X. (2015). A CRISPR/Cas9 toolbox for multiplexed plant genome editing and transcriptional regulation. *Plant Physiol.* 169 971–985. 10.1104/pp.15.00636 26297141 PMC4587453

[B35] Mayer R. Raventos D. Chua N. H. (1996). det1, cop1, and cop9 mutations cause inappropriate expression of several gene sets. *Plant Cell* 8 1951–1959. 10.1105/tpc.8.11.1951 8953766 PMC161326

[B36] McNellis T. W. Von Arnim A. G. Araki T. Komeda Y. Misera S. Deng X. W. (1994). Genetic and molecular analysis of an allelic series of cop1 mutants suggests functional roles for the multiple protein domains. *Plant Cell* 6 487–500. 10.1105/tpc.6.4.487 8205001 PMC160452

[B37] Meinke D. W. (1985). Embryo-lethal mutants of *Arabidopsis thaliana*: analysis of mutants with a wide range of lethal phases. *Theor. Appl. Genet.* 69 543–552. 10.1007/BF00251102 24254011

[B38] Meinke D. W. Sussex I. M. (1979b). Isolation and characterization of six embryo-lethal mutants of *Arabidopsis thaliana*. *Dev. Biol.* 72 62–72. 10.1016/0012-1606(79)90098-8 510781

[B39] Meinke D. W. Sussex I. M. (1979a). Embryo-lethal mutants of *Arabidopsis thaliana*: a model system for genetic analysis of plant embryo development. *Dev. Biol.* 72 50–61. 10.1016/0012-1606(79)90097-6 510780

[B40] Millar A. J. Straume M. Chory J. Chua N. H. Kay S. A. (1995). The regulation of circadian period by phototransduction pathways in *Arabidopsis*. *Science* 267 1163–1166. 10.1126/science.7855596 7855596

[B41] Misera S. Muller A. J. Weiland-Heidecker U. Jurgens G. (1994). The FUSCA genes of *Arabidopsis*: negative regulators of light responses. *Mol. Gen. Genet.* 244 242–252. 10.1007/BF00285451 8058035

[B42] Mustilli A. C. Fenzi F. Ciliento R. Alfano F. Bowler C. (1999). Phenotype of the tomato high pigment-2 mutant is caused by a mutation in the tomato homolog of DEETIOLATED1. *Plant Cell* 11 145–157. 10.1105/tpc.11.2.145 9927635 PMC144164

[B43] Nagato Y. Kitano H. Kamijima O. Kikuchi S. Satoh H. (1989). Developmental mutants showing abnormal organ differentiation in rice embryos. *Theor. Appl. Genet.* 78 11–15. 10.1007/BF00299746 24227023

[B44] Naito Y. Hino K. Bono H. Ui-Tei K. (2015). CRISPRdirect: software for designing CRISPR/Cas guide RNA with reduced off-target sites. *Bioinformatics* 31 1120–1123. 10.1093/bioinformatics/btu743 25414360 PMC4382898

[B45] Nishimura A. Aichi I. Matsuoka M. (2006). A protocol for Agrobacterium-mediated transformation in rice. *Nat. Protoc.* 1 2796–2802. 10.1038/nprot.2006.469 17406537

[B46] Osterlund M. T. Hardtke C. S. Wei N. Deng X. W. (2000). Targeted destabilization of HY5 during light-regulated development of *Arabidopsis*. *Nature* 405 462–466. 10.1038/35013076 10839542

[B47] Park J. Bae S. Kim J. S. (2015). Cas-Designer: a web-based tool for choice of CRISPR-Cas9 target sites. *Bioinformatics* 31 4014–4016. 10.1093/bioinformatics/btv537 26358729

[B48] Pepper A. Delaney T. Washburn T. Poole D. Chory J. (1994). *DET1*, a negative regulator of light-mediated development and gene expression in Arabidopsis, encodes a novel nuclear-localized protein. *Cell* 78 109–116. 10.1016/0092-8674(94)90577-0 8033202

[B49] Satoh N. Hong S. K. Nishimura A. Matsuoka M. Kitano H. Nagato Y. (1999). Initiation of shoot apical meristem in rice: characterization of four SHOOTLESS genes. *Development* 126 3629–3636. 10.1242/dev.126.16.3629 10409508

[B50] Schroeder D. F. Gahrtz M. Maxwell B. B. Cook R. K. Kan J. M. Alonso J. M. (2002). De-etiolated 1 and damaged DNA binding protein 1 interact to regulate *Arabidopsis* photomorphogenesis. *Curr. Biol.* 12 1462–1472. 10.1016/s0960-9822(02)01106-5 12225661

[B51] Seo J. Lee G. Jin Z. Kim B. Chin J. H. Koh H. J. (2020). Development and application of indica-japonica SNP assays using the Fluidigm platform for rice genetic analysis and molecular breeding. *Mol. Breed.* 40:39. 10.1007/s11032-020-01123-x

[B52] Sheridan W. F. Clark J. K. (1993). Mutational analysis of morphogenesis of the maize embryo. *Plant J.* 3 347–358. 10.1111/j.1365-313X.1993.tb00186.x

[B53] Shi H. Wang X. Mo X. Tang C. Zhong S. Deng X. W. (2015). *Arabidopsis* DET1 degrades HFR1 but stabilizes PIF1 to precisely regulate seed germination. *Proc. Natl. Acad. Sci. U.S.A.* 112 3817–3822. 10.1073/pnas.1502405112 25775589 PMC4378405

[B54] Stracke R. Favory J. J. Gruber H. Bartelniewoehner L. Bartels S. Binkert M. (2010). The *Arabidopsis* bZIP transcription factor HY5 regulates expression of the *PFG1/MYB12* gene in response to light and ultraviolet-B radiation. *Plant Cell Environ.* 33 88–103. 10.1111/j.1365-3040.2009.02061.x 19895401

[B55] Suzuki G. Yanagawa Y. Kwok S. F. Matsui M. Deng X. W. (2002). *Arabidopsis* COP10 is a ubiquitin-conjugating enzyme variant that acts together with COP1 and the COP9 signalosome in repressing photomorphogenesis. *Genes Dev.* 16 554–559. 10.1101/gad.964602 11877375 PMC155353

[B56] Wei N. Kwok S. F. Von Arnim A. G. Lee A. Mcnellis T. W. Piekos B. (1994). Arabidopsis COP8, COP10, and COP11 genes are involved in repression of photomorphogenic development in darkness. *Plant Cell* 6 629–643. 10.1105/tpc.6.5.629 8038603 PMC160464

[B57] Winters A. L. Minchin F. R. (2005). Modification of the Lowry assay to measure proteins and phenols in covalently bound complexes. *Anal. Biochem.* 346 43–48. 10.1016/j.ab.2005.07.041 16197913

[B58] Yanagawa Y. Sullivan J. A. Komatsu S. Gusmaroli G. Suzuki G. Yin J. (2004). *Arabidopsis* COP10 forms a complex with DDB1 and DET1 in vivo and enhances the activity of ubiquitin conjugating enzymes. *Genes Dev.* 18 2172–2181. 10.1101/gad.1229504 15342494 PMC515294

[B59] Yen H. C. Shelton B. A. Howard L. R. Lee S. Vrebalov J. Giovannoni J. J. (1997). The tomato high-pigment (hp) locus maps to chromosome 2 and influences plastome copy number and fruit quality. *Theor. Appl. Genet.* 95 1069–1079. 10.1007/s001220050664

[B60] Zang G. Zou H. Zhang Y. Xiang Z. Huang J. Luo L. (2016). The De-Etiolated 1 Homolog of Arabidopsis Modulates the ABA Signaling Pathway and ABA Biosynthesis in Rice. *Plant Physiol.* 171 1259–1276. 10.1104/pp.16.00059 27208292 PMC4902595

