# Supplementary material for: Mutations in OsDET1, OsCOP10, and OsDDB1 confer embryonic lethality and alter flavonoid accumulation in Rice (Oryza sativa L.) seed
Source: Front Plant Sci. 2022 Jul 25;13:952856. doi: 10.3389/fpls.2022.952856 (PMC9358687; doi:10.3389/fpls.2022.952856)
Supplement: Supplementary file 1 [file Table_1.DOCX]

Supplementary Material

# Supplementary Data

## Supplementary File 1. 3D visualization of the air space that exists between embryo and endosperm in SDJ (WT) (left) and *yel*-*sdj* mutant grain (right). Embryo (upper; yellow), air space (middle; blue), Endosperm (lower; white).

# Supplementary Figures and Tables

## Supplementary Table

**Supplementary Table 1.** **Information of the primers used in this study**

| **Oligo name** | **Forward primer (5’-3’)** | **Reverse primer (5’-3’)** | **Purpose** | **Note** |
| --- | --- | --- | --- | --- |
| S01002 | AAGGTGATGCTCCGGAATAA | ATGCGCACCTTACCTCTCTC | Mapping | InDel |
| RM3252 | ATGCAAGCATCTGCTTATGG | GTTGGTAACTTTGTTCCCATGC | Mapping | SSR |
| RM499 | CCGTACCAAACACCAACACTGC | CGGCCGATCATCTAATCTAATCTACC | Mapping | SSR |
| S01004 | GCATCCTGTCCCTCCTGATA | GCATGGTTTTGTGATATATTAATTTGG | Mapping | InDel |
| S01006 | TCAATCAAACCACGTTGCAT | CCCATGGCCTTAGCTTATTG | Mapping | InDel |
| S01011 | GCACATCACTGCAGATTTGG | GAACCAAGCTGGGAACTTTG | Mapping | InDel |
| *yel*-*sdj*_cs | TTTCCCCCTCTTGTTGACAG | AGATGGGACGGTACACGATT | Co-segregation analysis for yel-sdj | InDel |
| OsDET1_g1 | gtgtGAACTGGCGGTCGAAGACCC | aaacGGGTCTTCGACCGCCAGTTC | CRISPR/Cas9 construct |  |
| OsDET1_g2 | gtgtGGAAGATCATGAGAGTCGCA | aaacTGCGACTCTCATGATCTTCC | CRISPR/Cas9 construct |  |
| OsCOP10_g1 | gtgtgCGACTGCTCCGCCGGCCCTA | aaacTAGGGCCGGCGGAGCAGTCGc | CRISPR/Cas9 construct |  |
| OsDDB1_g1 | gtgtgAAGCAAGTGGATCTCGATAC | aaacGTATCGAGATCCACTTGCTTc | CRISPR/Cas9 construct |  |
| RT_OsDET1 | TGCAGCCAACTGTTGTCAAT | GCACCGCTCACAACTGTTAC | qRT-PCR |  |
| RT_OsCOP1 | GGGAGTGAAACGAATGAGGT | TCCAGCAGACTGCGCTAATA | qRT-PCR |  |
| RT_OsCOP10 | GCCGAGCATTGGACATTTAT | CCTATCTTGCAAACCTCATCG | qRT-PCR |  |
| RT_OsDDB1 | CACCCGTATCGAGATCCACT | GCGGAAGAGCTCTAGTGTGG | qRT-PCR |  |
| RT_OsACTIN-3 | AGGCAGTCAGTCAGATCACGA | GAGACATTCAATGCACCAGCA | qRT-PCR |  |
